# Association Between Transfusion Status, Hemoglobin Levels, and Patient‐Reported Outcomes in Myelofibrosis: A Post Hoc Clinical Trial Analysis

**DOI:** 10.1002/cam4.71729

**Published:** 2026-04-10

**Authors:** Ruben Mesa, Jeanne M. Palmer, Francesca Palandri, Lucia Masarova, Claire N. Harrison, Flora Mazerolle, Manal M'hari, Tom Liu, Shiyuan Zhang, Anna Cardellino, Zhaohui Wang, Catherine E. Ellis, Dwaipayan Patnaik, Antoine Regnault, Thomas W. LeBlanc

**Affiliations:** ^1^ Atrium Health Wake Forest Baptist Comprehensive Cancer Center Winston‐Salem North Carolina USA; ^2^ Mayo Clinic Phoenix Arizona USA; ^3^ IRCCS Azienda Ospedaliero‐ Universitaria di Bologna, Istituto di Ematologia, S. Orsola‐Malpighi Bologna Italy; ^4^ The University of Texas MD Anderson Cancer Center Houston Texas USA; ^5^ Guy's and St Thomas' NHS Foundation Trust London UK; ^6^ Modus Outcomes, a THREAD company Lyon France; ^7^ GSK Collegeville Pennsylvania USA; ^8^ GSK Baar Switzerland; ^9^ Duke University School of Medicine Durham North Carolina USA

**Keywords:** anemia, health‐related quality of life, hemoglobin, myelofibrosis, patient‐reported outcomes, symptoms

## Abstract

**Background:**

Quality of life and symptom burden of patients with myelofibrosis are well recognized and compounded in those with anemia; however, the effects of transfusion burden or anemia severity on quality of life have not been comprehensively characterized. This post hoc descriptive analysis explored the association between transfusion status or hemoglobin improvement and patient‐reported outcomes (PROs).

**Methods:**

The analysis used pooled populations across treatment arms from 3 clinical trials (SIMPLIFY‐1, SIMPLIFY‐2, MOMENTUM); sample sizes for each PRO measure were dependent on the trials in which they were administered.

**Results:**

At both baseline and week 24, transfusion independence was associated with umerically greater mean SF‐36v2 and EORTC QLQ‐C30 scores than transfusion dependence; in the subgroup that was transfusion dependent at baseline, those who achieved transfusion independence at week 24 had greater PRO improvements than those who remained reliant on transfusions. Regardless of transfusion status, patients who achieved a hemoglobin improvement ≥ 1, ≥ 1.5, or ≥ 2 g/dL from baseline also had clinically meaningful improvements in quality of life (assessed via mean EQ‐5D‐5L or SF‐36v2 scores) and symptoms (assessed via PGIC or MPN‐SAF/MFSAF Total Symptom Score) at week 24 compared with those who did not.

**Conclusions:**

Collectively, these results provide preliminary insights into the associations of transfusion status and anemia severity with quality of life in myelofibrosis; as current PRO measures do not directly evaluate the relationship between symptoms such as fatigue and anemia, development of new measures to more comprehensively capture the patient experience for those with anemia in myelofibrosis may be warranted.

**Trial Registration:**

NCT01969838, NCT02101268, NCT04173494

## 1 Introduction

Myelofibrosis is a chronic and progressive myeloproliferative neoplasm characterized by clinical manifestations including splenomegaly, cytopenias, and often debilitating symptom burden [1]. Symptom burden in myelofibrosis often translates into profound negative impacts on health‐related quality of life (HRQOL), compromising activities of daily living, social functioning, and work productivity [1, 2, 3, 4]. In addition to symptoms, HRQOL in myelofibrosis and other hematologic malignancies may be negatively impacted by factors such as disease risk, as higher risk may predict more aggressive disease progression and complications, emotional distress and other psychosocial factors, and financial burden [5, 6, 7, 8]. For these reasons, assessment of HRQOL is a common endpoint of myelofibrosis clinical trials. While several myelofibrosis‐specific patient‐reported outcome (PRO) measures have been developed to evaluate symptoms, no such measures exist for HRQOL in myelofibrosis, which is instead assessed using general measures not designed specifically for use in patients with myeloproliferative neoplasms [9, 10, 11].

HRQOL burden in myelofibrosis may be compounded in patients who have anemia, a key hallmark of the disease that often increases in prevalence and severity over time [12]. Anemia is one factor that contributes to fatigue in myelofibrosis, which is often reported by patients as their most frequent and debilitating symptom [1, 2, 13]. Other symptoms may also be exacerbated by anemia, and physical functioning and mental health impairments may further compromise activities of daily living [9]. As some Janus kinase (JAK) inhibitors may cause or worsen anemia while improving other disease manifestations, challenges in the treatment of anemia may also exacerbate HRQOL burden [13]. While red blood cell transfusions may help to alleviate anemia‐associated symptoms, they may also negatively impact HRQOL due to associated time and cost burdens as well as the risk of complications [13, 14, 15, 16, 17]. Both anemia and transfusion need are also negative prognostic factors in myelofibrosis [18, 19], suggesting that reduced transfusion burden and other anemia‐related improvements may positively impact both patient HRQOL and survival. While achieving improvement in anemia has been linked to improved PROs [20], the impact of transfusion dependence on HRQOL in myelofibrosis has not been comprehensively characterized. Furthermore, symptom and HRQOL improvements associated with treatment to improve anemia or reduce transfusion burden have not been described.

Momelotinib is a JAK1/JAK2/activin A receptor type 1 inhibitor approved for the treatment of patients with myelofibrosis and anemia [21, 22]. In 3 phase 3 trials across JAK inhibitor–naive and –experienced patients, momelotinib demonstrated anemia‐related benefits, including increased hemoglobin levels and transfusion independence rates at week 24, as well as symptom and splenomegaly improvements [23, 24, 25]. Each of these trials included a number of PRO measures to comprehensively evaluate HRQOL, although only limited PRO results beyond symptom improvement have been reported to date [23, 24, 25]. While not prospectively designed to evaluate the potential association between anemia improvement and HRQOL, inclusion of both anemia‐related benefits and multiple PRO measures as endpoints enables preliminary assessment of this relationship. To that end, we conducted an exploratory post hoc analysis of 3 phase 3 trials to evaluate the association between transfusion status, anemia, and the patient experience in myelofibrosis, using PROs to capture concepts such as symptom burden, HRQOL, and functioning.

## 2 Materials and Methods

### 2.1 Study Design

The overall pooled analysis set comprised both arms of the intent‐to‐treat populations from each of the 3 global, randomized, phase 3 trials of momelotinib in adult patients with primary or secondary myelofibrosis [23, 24, 25]; specific analysis sets by PRO measure are described in the *Statistical Analysis* section. All 3 trials were conducted in accordance with the Declaration of Helsinki and the International Council for Harmonisation guidelines on Good Clinical Practice. The institutional review board or independent ethics committee at each study site approved the protocol, and all participants provided written informed consent.

Full study designs and primary results from each trial have been previously reported [23, 24, 25]. SIMPLIFY‐1 (NCT01969838) was a double‐blind trial of momelotinib vs. ruxolitinib in JAK inhibitor–naive patients (*N* = 432) [23]. SIMPLIFY‐2 (NCT02101268) was an open‐label trial of momelotinib vs. best available therapy (88.5% ruxolitinib) in JAK inhibitor–experienced patients (*N* = 156) [25]. MOMENTUM (NCT04173494) was a double‐blind trial of momelotinib vs. danazol in JAK inhibitor–experienced patients with symptomatic (Total Symptom Score [TSS] ≥ 10) and anemic (hemoglobin < 10 g/dL) myelofibrosis at baseline (*N* = 195) [24]. The randomized treatment period of each trial was 24 weeks [23, 24, 25].

### 2.2 PRO Measures: HRQOL

The PRO measures included in these analyses are summarized in Table S1, which details the measures administered in each momelotinib clinical trial and the group‐level meaningful change thresholds (MCTs) applied.

#### 2.2.1 EQ‐5D‐5L

The EQ‐5D‐5L is a general (i.e., not disease‐specific) questionnaire in 2 parts [1]: a descriptive system rating 5 dimensions across 5 levels and [2] a single visual analog scale (VAS). Each dimension of the descriptive system is rated on a 5‐point scale from 1 (no problem) to 5 (extreme problem) on the day administered. The rating for each dimension represents 1 digit of a 5‐digit code corresponding to overall health state, weighted against utility value sets reflecting the preferences of a population (UK: SIMPLIFY‐1 and SIMPLIFY‐2; US: MOMENTUM) [26, 27] to derive a single index score reflecting how good or bad that health state is (1 = perfect health). The VAS rates general health from 0 (worst imaginable) to 100 (best imaginable). Assessment of PROs via the EQ‐5D‐5L was an exploratory endpoint of all 3 trials, administered at baseline and then every 4 weeks in SIMPLIFY‐1 and SIMPLIFY‐2 and at baseline, week 12, and week 24 in MOMENTUM [23, 24, 25]. Changes > 0.037 in index scores and > 7 in VAS scores were considered clinically meaningful (Table S1) [27, 28].

#### 2.2.2 SF‐36v2

The 36‐Item Short Form Survey, version 2 (SF‐36v2), is a general measure of patients' self‐reported health, well‐being, and functioning across 8 HRQOL domains, with high scores indicating better HRQOL and/or functioning; norm‐based score ranges vary by domain, with a mean score of 50 (standard deviation [SD], 10) corresponding to the US general population [29, 30]. Assessment of PROs via the SF‐36v2 was an exploratory endpoint in both SIMPLIFY trials, completed at baseline and then every 4 weeks on study to week 24 [23, 25]. The recall period for all domains was 4 weeks, except for the physical functioning and general health domains, which assessed health at the time of administration. MCTs varied by domain from 2 to 4 (Table S1) [30].

#### 2.2.3 EORTC QLQ‐C30

The European Organisation for Research and Treatment of Cancer Quality of Life Questionnaire–Core 30 (EORTC QLQ‐C30) is a self‐report measure of HRQOL in patients with cancer, with 30 items spanning 15 domains covering global health status, symptoms, and functioning; scores are transformed on a scale from 0 to 100, with high scores indicating either better global health status/functioning or worse symptoms, and meaningful changes vary by domain [31, 32]. Assessment of cancer‐related fatigue via the EORTC QLQ‐C30 fatigue domain was a secondary endpoint in MOMENTUM; assessment of PROs via other EORTC QLQ‐C30 domains was an exploratory endpoint. In MOMENTUM, the EORTC QLQ‐C30 was completed at baseline, week 12, and week 24 [24]. The recall period was 7 days for all domains; responses for most items ranged from 1 (not at all) to 4 (very much), while those for the 2 items composing the global quality of life domain ranged from 1 (very poor) to 7 (excellent). MCTs varied by domain from 7 to 13 (Table S1) [32].

### 2.3 PRO Measures: Symptoms

#### 2.3.1 MPN‐SAF v2.0/MFSAF v4.0

Both the Myeloproliferative Neoplasm Symptom Assessment Form (MPN‐SAF) and Myelofibrosis Symptom Assessment Form (MFSAF) are disease‐specific measures that score the severity of 7 myelofibrosis‐related symptoms over the past 24 h [10, 11]. Scores for each item range from 0 (absent) to 10 (worst imaginable); therefore, the combined TSS has a maximum (worst) of 70. A version of the TSS was administered daily from baseline in all 3 trials: MPN‐SAF v2.0 was used in SIMPLIFY‐1 and SIMPLIFY‐2 (TSS reduction ≥ 50% at week 24 was a key secondary endpoint), and MFSAF v4.0 was used in MOMENTUM (TSS reduction ≥ 50% at week 24 was the primary endpoint); baseline TSS was the daily average of the prior 7 days, and week 24 TSS was the daily average of the prior 28 days (≥ 20 days with nonmissing scores) [23, 24, 25]. Based on the MFSAF v4.0, a change in absolute TSS of 1.5 was considered clinically meaningful (Table S1) [33].

#### 2.3.2 PGIC

The Patient Global Impression of Change (PGIC) is a general item that asks a single question about myelofibrosis‐related symptom improvement since the start of treatment, with scores ranging from 1 (very much improved) to 7 (very much worse). The PGIC item used in these analyses was administered every 2 weeks in SIMPLIFY‐1 and SIMPLIFY‐2 to assess symptoms as an exploratory endpoint of both trials [23, 25]. Changes were classified as improved (scores of 1, 2, or 3), no change (score of 4), or worsened (scores of 5, 6, or 7) (Table S1).

### 2.4 Statistical Analysis

The analysis set for each PRO measure was based on the trial(s) in which that assessment was administered; results may represent the pooled SIMPLIFY‐1/SIMPLIFY‐2/MOMENTUM population, the pooled SIMPLIFY‐1/SIMPLIFY‐2 population, or MOMENTUM alone. For PRO measures with multiple domains, results were analyzed in those domains hypothesized to have the most potential to be associated with anemia and/or transfusion burden, such as those related to physical functioning, social functioning/role‐emotional, and vitality/fatigue.

#### 2.4.1 Transfusion Status

Mean scores at baseline and week 24 and mean change from baseline to week 24 were summarized for the SF‐36v2 and EORTC QLQ‐C30 in subgroups of the intent‐to‐treat population defined by transfusion status regardless of baseline anemia. Transfusion status at each time point was defined as follows: transfusion independent (TI), no transfusions and all hemoglobin levels ≥ 8 g/dL in the previous 12 weeks (all 3 trials); TD, ≥ 4 red blood cell units transfused or a hemoglobin level < 8 g/dL in the previous 8 weeks (SIMPLIFY‐1/SIMPLIFY‐2) or ≥ 4 red blood cell units transfused, each associated with a hemoglobin level ≤ 9.5 g/dL, in the previous 8 weeks (MOMENTUM); or transfusion requiring (TR), not meeting the criteria for either TI or TD (all 3 trials). Results were summarized descriptively with potentially significant associations indicated by mean changes from baseline that exceeded the identified MCTs for each PRO measure and domain (Table S1).

#### 2.4.2 Hemoglobin Improvement

PROs based on hemoglobin improvement at week 24 were assessed in patients with anemia (hemoglobin < 10 g/dL) at baseline (*n* = 480; SIMPLIFY‐1, *n* = 180; SIMPLIFY‐2, *n* = 105; MOMENTUM, *n* = 195). Laboratory assessments were performed at baseline and every 2 weeks through week 24 in SIMPLIFY‐1 and SIMPLIFY‐2 and at baseline, week 2, week 4, and every 4 weeks thereafter through week 24 in MOMENTUM [23, 24, 25]. Hemoglobin improvement was defined as an increase of ≥ 1, ≥ 1.5, or ≥ 2 g/dL from baseline at week 24 [34, 35, 36].

Assessments included changes from baseline in EQ‐5D‐5L index and VAS scores, selected SF‐36v2 domain scores (physical functioning, role‐physical, mental health, social functioning, and vitality), and absolute MPN‐SAF or MFSAF TSS, as well as the percentages of patients with improved, no change in, or worsening symptoms per PGIC, for subgroups that did vs. those that did not achieve each hemoglobin improvement threshold. To confirm potentially significant associations based on identified MCTs for each PRO measure and domain (Table S1), multivariate linear regression models were derived that included change in EQ‐5D‐5L score (either index or VAS) or absolute MPN‐SAF/MFSAF TSS from baseline at week 24 as the dependent variable, and week 24 hemoglobin improvement and key baseline characteristics of potential clinical relevance that were available for the majority of patients across trials (age, sex, race, geographic region, myelofibrosis subtype, *JAK2* V617F status, platelet count, and baseline score) as independent variables; statistical testing was not controlled for multiplicity and no interactions between potential covariates were evaluated.

## 3 Results

### 3.1 Association of Transfusion Status With PROs

Baseline characteristics of the pooled analysis sets for transfusion status (SIMPLIFY‐1/SIMPLIFY‐2 [*n* = 588] and MOMENTUM [*n* = 195]) are shown in Table 1. Consistent with inclusion of JAK inhibitor–naive patients from SIMPLIFY‐1, the pooled SIMPLIFY‐1/SIMPLIFY‐2 analysis set had fewer patients with severe anemia (hemoglobin < 8 g/dL; 13.9% vs. 48.2%) and more patients who were TI (59.5% vs. 13.8%) at baseline than the MOMENTUM analysis set.

**TABLE 1:** Baseline characteristics of the analysis sets for transfusion status.

Baseline characteristics^a^	Pooled SIMPLIFY‐1 + SIMPLIFY‐2 (*n* = 588)	MOMENTUM (*n* = 195)
Age, median (range), years	67 (25–92)	71 (38–86)
Male sex at birth, *n* (%)	337 (57.3)	123 (63.1)
White, *n* (%)	484 (82.3)	157 (80.5)
MF subtype, *n* (%)		
PMF	338 (57.5)	124 (63.6)
PET‐MF	122 (20.7)	33 (16.9)
PPV‐MF	128 (21.8)	38 (19.5)
Risk level, *n* (%)^b^		
Intermediate‐1	128 (21.8)	10 (5.1)
Intermediate‐2	233 (39.6)	112 (57.4)
High	227 (38.6)	69 (35.4)
Palpable spleen size, median (range), cm	NA	11 (2–39)
Platelet count, *n* (%)		
< 100 × 10^9^/L	101 (17.2)	100 (51.3)
≥ 100 to ≤ 200 × 10^9^/L	178 (30.3)	50 (25.6)
> 200 × 10^9^/L	298 (76.8)	42 (21.5)
Hb level, *n* (%)		
< 8 g/dL	82 (13.9)	94 (48.2)
≥ 8 g/dL	505 (85.9)	100 (51.3)
Transfusion status, *n* (%)		
TI	350 (59.5)	27 (13.8)
TD	190 (32.3)	97 (49.7)
TR	48 (8.2)	71 (36.4)
*JAK2* V617F mutation status, *n* (%)		
Positive	373 (63.4)	148 (75.9)
Negative	157 (26.7)	40 (20.5)

Abbreviations: DIPSS, Dynamic International Prognostic Scoring System; Hb, hemoglobin; IPSS, International Prognostic Scoring System; JAK, Janus kinase; MF, myelofibrosis; NA, not available; PET‐MF, post–essential thrombocythemia myelofibrosis; PMF, primary myelofibrosis; PPV‐MF, post–polycythemia vera myelofibrosis; TD, transfusion dependent; TI, transfusion independent; TR, transfusion requiring.

^a^ Some categories may not total 100% due to patients with missing data.

^b^ Per IPSS in SIMPLIFY‐1 and SIMPLIFY‐2 and per DIPSS in MOMENTUM.

**FIGURE 1:**
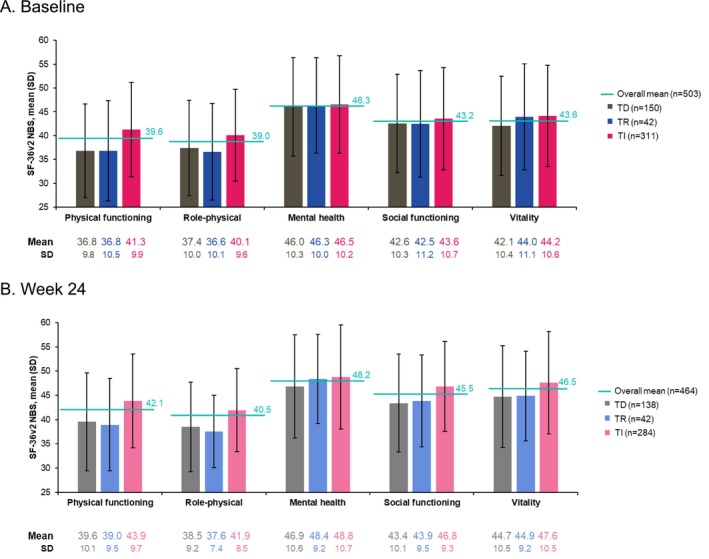
SF‐36v2 scores at baseline (A) and week 24 (B) by transfusion status at each time point in SIMPLIFY‐1 and SIMPLIFY‐2. Higher scores indicate improvement for all domains. NBS, norm‐based score; SD, standard deviation; SF‐36v2, 36‐Item Short Form Survey, version 2; TD, transfusion dependent; TI, transfusion independent; TR, transfusion requiring.

SF‐36v2 results were available from 503 patients in the pooled SIMPLIFY trials at baseline; across all domains evaluated (physical functioning, role‐physical, mental health, social functioning, and vitality), overall mean baseline scores in this clinical trial population with myelofibrosis were numerically lower than the reported general population mean of 50 (Figure 1A) [30]. Patients who were TD or TR at baseline had numerically lower mean scores than those who were TI across the domains of interest; differences between TD and TI patients exceeded the predefined MCTs for the physical functioning (mean, 36.8 vs. 41.3; MCT, 3), role‐physical (mean, 37.4 vs. 40.1; MCT, 3), and vitality (mean, 42.1 vs. 44.2; MCT, 2) domains (Figure 1A).

At week 24, SF‐36v2 results were available from 464 patients, and trends were similar to those observed at baseline (Figure 1B); while mean SF‐36v2 scores overall remained numerically lower than the general population mean of 50, patients who were TD or TR at week 24 had even lower mean SF‐36v2 scores than those who were TI across all domains. Associations between transfusion status and PROs in the JAK inhibitor–experienced population of MOMENTUM were also observed in comparable domains of the EORTC QLQ‐C30 at both baseline and week 24 (Figure S1). The differences between patients who were TD vs. those who were TI exceeded the predefined MCTs indicating worse physical functioning at baseline (mean, 50.9 vs. 63.3; MCT, 7) and increased fatigue at both baseline and week 24 (baseline mean, 65.7 vs. 51.4; week 24 mean, 54.8 vs. 43.8; MCT, 9) in TD patients.

**FIGURE 2:**
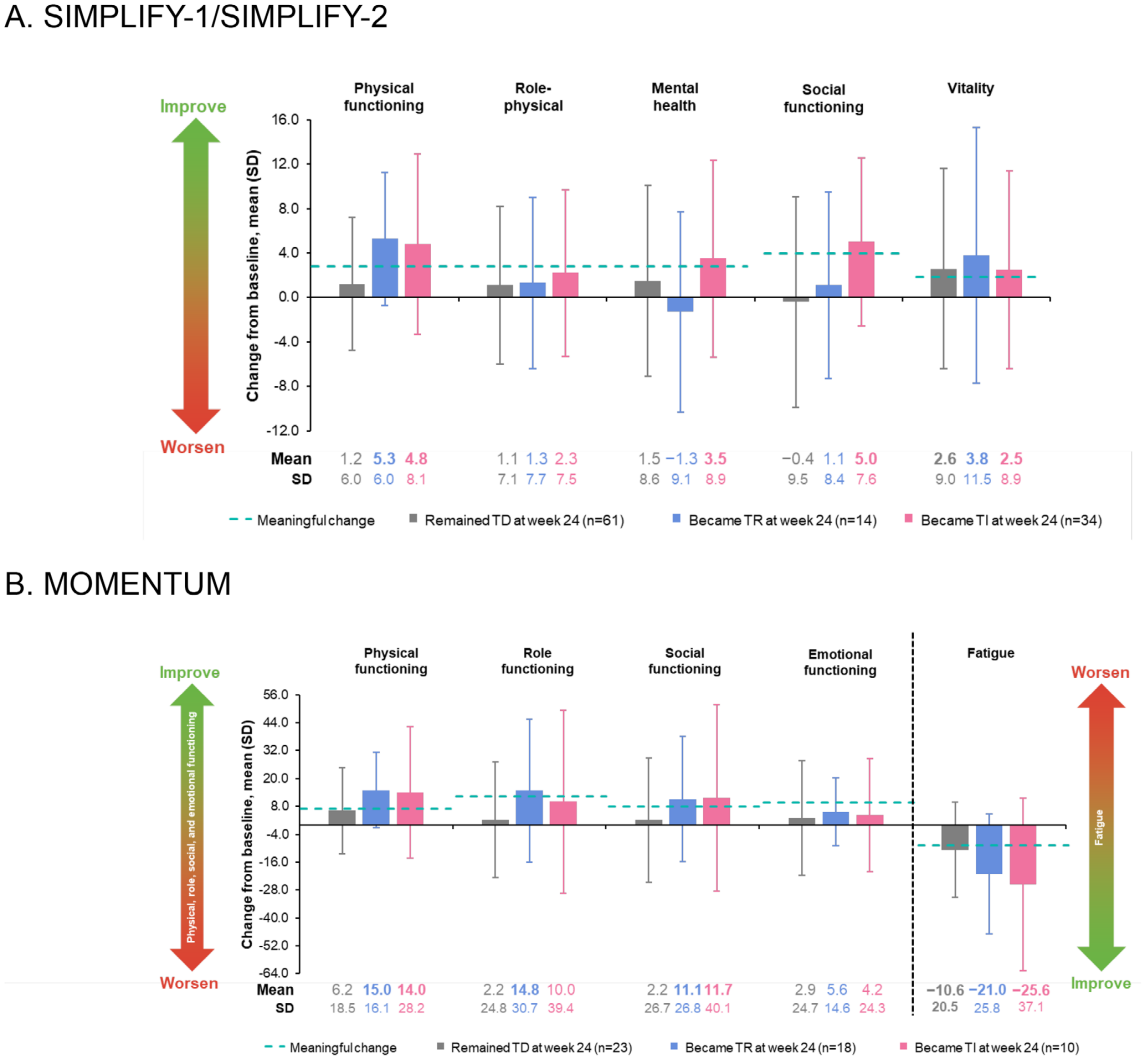
Mean change from baseline at week 24 in SF‐36v2 scores in the baseline TD patient subgroup in SIMPLIFY‐1 and SIMPLIFY‐2 (A) and in mean EORTC QLQ‐C30 scores in the baseline TD patient subgroup in MOMENTUM (B). Bolding indicates mean changes from baseline that exceeded the MCT for the domain (SF36v2: 2.0 for vitality, 4.0 for role‐emotional, 3.0 for remaining domains; EORTC QLQ‐C30: 7 for physical functioning, 8 for social functioning, 9 for emotional functioning and fatigue, 12 for role‐functioning). As indicated by the dashed black line, direction of improvement is opposite for fatigue vs. the other EORTC QLQ‐C30 domains shown. EORTC QLQ‐C30, European Organisation for Research and Treatment of Cancer Quality of Life Questionnaire–Core 30; MCT, meaningful change threshold; SD, standard deviation; SF‐36v2, 36‐Item Short Form Survey, version 2; TD, transfusion dependent; TI, transfusion independent; TR, transfusion requiring.

Among patients in the pooled SIMPLIFY trials who were TD and had SF‐36v2 results at baseline, 75 remained TD (50.0%), 21 became TR (14.0%), 40 became TI (26.7%), and 14 had no data (9.3%) at week 24 (Figure 2A and Figure S2A). Patients who were TD at baseline but became TI at week 24 experienced numerically greater improvement (positive mean change from baseline vs. those who remained TD) in all assessed SF‐36v2 domains except vitality; mean changes from baseline in patients who became TI exceeded the MCT for all SF‐36v2 domains except role‐physical. Mean changes from baseline (TI vs. TD) were 4.8 vs. 1.2 for the physical functioning, 2.2 vs. 1.1 for the role‐physical, 3.5 vs. 1.5 for the mental health (MCT, 3); 5.0 vs. −0.4 for the role‐emotional (MCT, 4); and 2.5 vs. 2.6 for the vitality (MCT, 2) domains. While the sample size without pooling was small, generally consistent trends were observed in the individual SIMPLIFY‐1 and SIMPLIFY‐2 study populations, although JAK inhibitor–naive patients were more likely to achieve meaningful change (Table S2). Similar trends were also observed in comparable domains of the EORTC QLQ‐C30 for baseline TD patients at week 24 in MOMENTUM (Figure 2B and Figure S2B).

### 3.2 Association of Hemoglobin Improvement With PROs

At both baseline and week 24 in the SIMPLIFY trials, a modest incremental effect of hemoglobin level on the SF‐36v2 physical functioning domain was observed; no clear association was found between hemoglobin levels and other SF‐36v2 domains (data not shown). To further evaluate the association between hemoglobin level and PROs, the analysis was focused on hemoglobin improvement at week 24 in the pooled population of patients with anemia at baseline (hemoglobin < 10 g/dL; SIMPLIFY‐1, SIMPLIFY‐2, and MOMENTUM) (Table S3). Patients achieving hemoglobin improvement at each threshold are summarized in Table S4 for each pooled analysis set (all 3 trials, SIMPLIFY‐1/SIMPLIFY‐2, or MOMENTUM only), dependent on in which trial(s) a given PRO measure was administered. A total of 436 of 480 patients (90.8%) were evaluable for hemoglobin improvement at week 24 across all 3 trials: 241 of 285 patients with anemia (84.6%) in SIMPLIFY‐1/SIMPLIFY‐2 and all 195 patients in MOMENTUM. Nearly 50% of patients achieved hemoglobin improvement of ≥ 1 g/dL in each analysis set, while 16% to 26% achieved improvement of ≥ 2 g/dL.

**FIGURE 3:**
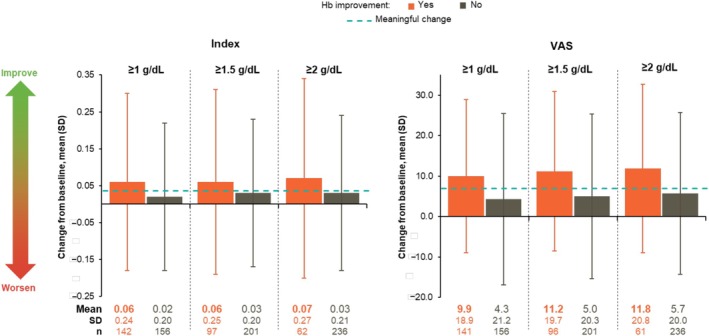
Mean change from baseline in EQ‐5D‐5L index (A) and VAS (B) scores at week 24 based on hemoglobin improvement in all 3 trials. *n* = 298 for index scores; *n* = 297 for VAS scores. Bolding indicates mean changes from baseline that exceeded the MCT (0.037 for index, 7 for VAS). Hb, hemoglobin; MCT, meaningful change threshold; SD, standard deviation; VAS, visual analog scale.

Changes from baseline in EQ‐5D‐5L index and VAS scores were available for 298 and 297 patients, respectively, from all 3 trials (Figure 3). Mean improvements were clinically meaningful and numerically greater in patients who achieved hemoglobin improvement at any threshold than in those who did not. Mean changes from baseline in index scores ranged from 0.06 to 0.07 for patients who achieved varying hemoglobin improvement thresholds vs. 0.02 to 0.03 for those who did not (MCT, 0.037). Mean changes from baseline in VAS scores ranged from 9.9 to 11.8 vs. 4.3 to 5.7 (MCT, 7). While sample sizes without pooling were small, EQ‐5D‐5L index and VAS score changes in the individual SIMPLIFY‐1, SIMPLIFY‐2, and MOMENTUM study populations exceeded the MCTs across all hemoglobin improvement thresholds; notably, patients who did not achieve hemoglobin improvement in SIMPLIFY‐1 had a meaningful worsening in index scores (Table S5). In multivariate regression analyses not controlled for multiplicity, hemoglobin improvement at any threshold was statistically significantly associated with positive change from baseline in EQ‐5D‐5L VAS scores at week 24; similar but nonsignificant trends were observed for EQ‐5D‐5L index scores (Table 2 and Table S6).

**TABLE 2:** Multivariate analysis for change from baseline in EQ‐5D‐5L VAS scores (dependent variable) with hemoglobin improvement at week 24 and baseline characteristics as independent variables (pooled SIMPLIFY‐1, SIMPLIFY‐2, MOMENTUM).

Hb improvement (*n* = 297)		≥ 1 g/dL (yes, *n* = 141; no, *n* = 156)	≥ 1.5 g/dL (yes, *n* = 96; no, *n* = 201)	≥ 2 g/dL (yes, *n* = 61; no, *n* = 236)
Independent variable	Category	Parameter estimates		
Hb improvement (ref: no)	Yes	**5.80** *	**5.27** *	**6.84** *
Age (continuous)	—	0.12	0.11	0.08
Sex (ref: male)	Female	1.37	1.23	1.13
Race (ref: Black)	Asian Not reported Other White	−29.37 −12.50 −11.47 −16.73	−30.35 −13.56 −11.47 −17.22	**−32.58** * −14.38 −13.21 **−18.30** *
Region (ref: North America)	Asia Australasia Eastern Europe Western Europe	10.84 4.70 −1.18 −2.71	11.41 4.41 −1.18 −2.78	12.05 3.86 −1.14 −2.77
MF subtype (ref: primary)	PET PPV	**−5.98** * −4.92	−6.28 −4.40	−6.85 −4.66
*JAK2* V617F mutation status (ref: negative)	Positive Unknown	0.07 −3.65	−0.28 −5.00	−0.20 −5.62
Baseline platelet count (ref: ≤ 150 × 10^9^/L)	> 150 × 10^9^/L	0.53	0.26	0.37
Baseline VAS score (continuous)	—	**−0.56** *	**−0.55** *	**−0.55** *

*Note:* Parameter estimates are the predicted changes from baseline based on a given independent variable after controlling for all other independent variables. Age and baseline score were assessed as continuous variables; all others were categorical vs. the indicated reference (ref) categories. Bold numbers with * indicate statistically significant changes from baseline (*p* < 0.05).

Abbreviations: Hb, hemoglobin; JAK, Janus kinase; MF, myelofibrosis; PET, post–essential thrombocythemia; PPV, post–polycythemia vera; VAS, visual analog scale.

**FIGURE 4:**
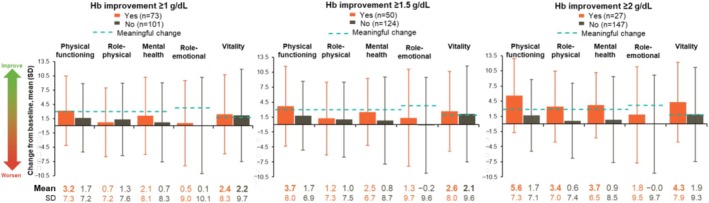
Mean change from baseline in SF‐36v2 domains at week 24 based on hemoglobin improvement in SIMPLIFY‐1 and SIMPLIFY‐2. *n* = 174. Bolding indicates mean changes from baseline that exceeded the MCT for the domain (2.0 for vitality, 4.0 for role‐emotional, 3.0 for remaining domains). Hb, hemoglobin; MCT, meaningful change threshold; SD, standard deviation; SF‐36v2, 36‐Item Short Form Survey, version 2.

Changes from baseline in SF‐36v2 domains were available for 174 patients from SIMPLIFY‐1/SIMPLIFY‐2 (Figure 4). Mean improvements from baseline were numerically greater in patients who achieved hemoglobin improvement than in those who did not across all domains, with the exception of role‐physical in the hemoglobin improvement ≥ 1 g/dL group only. Mean improvements from baseline in the physical functioning and vitality domains consistently exceeded the MCT in patients who achieved hemoglobin improvement at any threshold. Changes from baseline in the physical functioning domain in patients with hemoglobin improvement ranged from 3.2 to 5.6 (MCT, 3); changes from baseline for patients who did not achieve hemoglobin improvement did not reach the threshold for meaningful change. Changes from baseline in the vitality domain in patients with hemoglobin improvement ranged from 2.4 to 4.3 (MCT, 2); however, changes from baseline for patients who did not achieve hemoglobin improvement (≥ 1‐g/dL or ≥ 1.5‐g/dL thresholds) were also meaningful.

**FIGURE 5:**
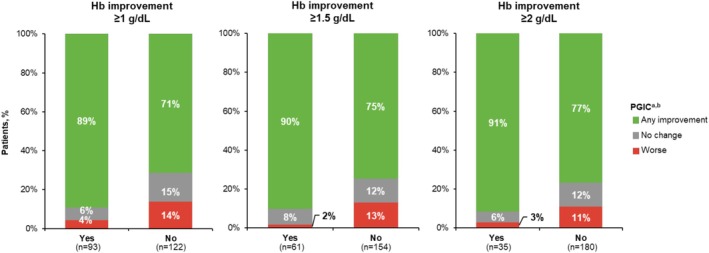
Mean change from baseline in symptoms at week 24 by PGIC based on hemoglobin improvement in SIMPLIFY‐1 and SIMPLIFY‐2. Hb, hemoglobin; PGIC, Patient Global Impression of Change.^a^ “Any improvement” corresponds to PGIC scores of 1 (“very much improved”), 2 (“much improved”), or 3 (“minimally improved”). “No change” corresponds to a PGIC score of 4. “Worse” corresponds to PGIC scores of 5 (“minimally worse”), 6 (“much worse”), or 7 (“very much worse”).^b^ Patients with missing data were excluded from the analysis and are not reflected in the percentages shown.

**FIGURE 6:**
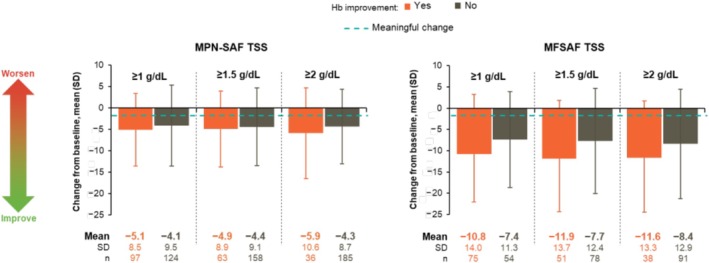
Mean absolute change from baseline in MF‐related symptoms at week 24 by MPN‐SAF v2.0 in SIMPLIFY‐1 and SIMPLIFY‐2 (left; *n* = 221) and by MFSAF v4.0 in MOMENTUM (right; *n* = 129). Bolding indicates mean changes from baseline that exceeded the MCT (1.5). Hb, hemoglobin; MCT, meaningful change threshold; MF, myelofibrosis; MFSAF, Myelofibrosis Symptom Assessment Form; MPN‐SAF, Myeloproliferative Neoplasm Symptom Assessment Form; SD, standard deviation; TSS, Total Symptom Score.

PGIC at week 24 was available for 215 patients with baseline anemia in SIMPLIFY‐1/SIMPLIFY‐2 (Figure 5). Hemoglobin improvement at any threshold was associated with numerically higher percentages of patients with any symptom improvement (89% to 91% vs. 71% to 77%) and lower percentages with symptom worsening (2% to 4% vs. 11% to 14%). Further analysis of symptom improvement was available for the 221 patients with anemia in SIMPLIFY‐1/SIMPLIFY‐2 and 129 patients in MOMENTUM who had full MPN‐SAF and MFSAF, respectively, TSS data available through week 24 (Figure 6). Clinically meaningful mean decreases (improvement) in absolute TSS were observed in all patients regardless of hemoglobin improvement, ranging from 4.1 to 5.9 for the pooled SIMPLIFY‐1/SIMPLIFY‐2 population (MPN‐SAF) and 7.4 to 11.9 in the baseline symptomatic population of MOMENTUM (MFSAF) (MCT, 1.5). However, changes were numerically greater in patients who achieved hemoglobin improvement at any threshold than in those who did not in both SIMPLIFY‐1 and SIMPLIFY‐2 (4.9 to 5.9 vs. 4.1 to 4.4) and MOMENTUM (10.8 to 11.9 vs. 7.4 to 8.4), although these differences were less pronounced in the SIMPLIFY analysis. In multivariate analyses not controlled for multiplicity, hemoglobin improvement at any threshold was associated with positive change from baseline in absolute TSS at week 24, but these trends generally did not reach the level of statistical significance; only hemoglobin improvement of ≥ 1 g/dL in MOMENTUM was significantly associated with absolute TSS improvement (Tables S7 and S8).

## 4 Discussion

In this clinical trial population of patients with myelofibrosis, achieving either transfusion independence or hemoglobin improvement of any threshold at week 24 was associated with clinically meaningful improvements in symptoms (as assessed by MPN‐SAF/MFSAF TSS and PGIC) and aspects of HRQOL, particularly domains related to physical functioning and fatigue (as assessed by multiple general HRQOL measures). As illustrated by SF‐36v2 scores at baseline, the overall patient population with myelofibrosis had lower mean HRQOL than the general population, and this burden was compounded in patients with transfusion dependence. Multivariate analyses further highlighted hemoglobin improvement at week 24 as a clinically significant variable associated with improvements in HRQOL measured by EQ‐5D‐5L VAS, when adjusted for baseline demographic and clinical characteristics. Previous studies have assessed hemoglobin improvement at varying thresholds, with recently updated International Working Group‐Myeloproliferative Neoplasms Research and Treatment anemia response guidelines proposing a cutoff of 1 g/dL for minor response and 1.5 g/dL for major response [34, 35, 36]. Our analyses suggest that even a minor anemia response may be associated with clinically meaningful improvement in general HRQOL and physical functioning, pending prospective validation.

In addition to the momelotinib clinical trials included in the present analysis [23, 24, 25], clinical trials of the approved JAK inhibitors ruxolitinib [37, 38], fedratinib [39, 40], and pacritinib [41, 42] all include multiple PRO measures. With the exception of the COMFORT‐II trial of ruxolitinib, all of these studies include myelofibrosis‐related symptom evaluation via the MPN‐SAF or MFSAF as a primary or secondary endpoint [23, 24, 25, 37, 38, 39, 40, 41, 42]. EORTC QLQ‐C30, EQ‐5D‐5L, and PGIC are frequently included as secondary or exploratory endpoints to evaluate general HRQOL and symptoms [23, 24, 25, 37, 38, 39, 40, 41, 42]. However, associations between transfusion status, anemia severity, and improvements in these PRO measures in myelofibrosis have been limited to date. A phase 2 study of pomalidomide in patients with myelofibrosis and anemia found a correlation between anemia response and improved HRQOL, as assessed using the anemia subscale of the Functional Assessment of Cancer Therapy measure (FACT‐An) [20]. The present analyses expand on this previously limited evidence linking anemia and PROs in myelofibrosis.

Beyond providing new insights into the association between anemia and the patient experience in myelofibrosis, these analyses also highlight the challenges of assessing this association in the clinical trial setting. The diversity of general HRQOL measures included across myelofibrosis clinical trials is in part attributable to the fact that none of these measures are specific to disease state. The SF‐36v2, EORTC QLQ‐C30, and EQ‐5D‐5L are general PRO measures and do not specifically characterize the experience of patients with myelofibrosis and anemia and/or those receiving transfusions and thus may fail to fully capture changes specific to this disease state [28, 30, 31]. Similarly, the present analysis applies MCTs to these measures that were largely not derived from myelofibrosis datasets; thus, it is difficult to determine definitively if the observed changes are meaningful to patients with myelofibrosis [27, 28, 30, 32]. The administration schedule of these measures in the trial setting is also challenging, as assessment every 4 or 12 weeks may not be sufficiently granular to capture changes due to transfusion status or anemia severity. Transfusions themselves represent a potential confounding factor in analyses of HRQOL in the context of myelofibrosis clinical trials, in the absence of predefined intercurrent event strategies to manage their effects [43, 44].

Although the MPN‐SAF and MFSAF are myelofibrosis‐specific measures [10, 11], they may not be sensitive enough to fully characterize the impact of anemia on myelofibrosis‐related symptoms, as patients who did not achieve hemoglobin improvement also showed meaningful absolute change in TSS from baseline in the present analysis. This may reflect potentially confounding factors such as receiving transfusions, as well as other positive effects of treatment such as spleen and symptom improvements. In addition, while these measures capture the myelofibrosis‐related symptoms that are most relevant to patients, they include those that are considered more attributable to splenomegaly (e.g., abdominal discomfort) or general constitutional malaise (e.g., weight loss) rather than a likely association with anemia [11]. Although the FACT‐An is an anemia‐specific measure previously applied to patients with myelofibrosis (although not in the momelotinib clinical trials discussed in the present analyses) [20], it was broadly designed for patients with cancer, primarily focuses on anemia‐related fatigue, and may yield variable results given that it combines several different FACT components into one measure [45]. Based on the preliminary evidence reported here on the association between anemia and HRQOL, these findings suggest that the development of new measures may be needed to improve the evaluation of changes in HRQOL associated with anemia and transfusion status in myelofibrosis.

The present analyses focus on pooled analysis sets across treatment arms, both to increase sample size and because the goal was to evaluate the association between transfusion status, anemia severity, and PROs, not to determine the effects of a particular treatment on these outcomes. Previous analyses have characterized the symptom and HRQOL benefits of the JAK inhibitors ruxolitinib and fedratinib in detail; however, these studies did not correlate these benefits with anemia‐related changes, perhaps in part due to the myelosuppressive profiles of both ruxolitinib and fedratinib [46, 47]. A previous post hoc analysis focused on the phase 3 MOMENTUM trial characterized the relative benefits of momelotinib vs. danazol in patients with myelofibrosis and anemia (hemoglobin < 10 g/dL) using both the MFSAF and general EORTC QLQ‐C30 and Patient‐Reported Outcomes Measurement Information System measures [9]. Momelotinib was associated with significant benefits vs. danazol across multiple measures and domains, notably in measures related to both cancer‐related and myelofibrosis‐related fatigue [9]. However, correlation of these improvements with anemia improvement was limited, and association between fatigue responses and transfusion independence was not definitive given the multifactorial nature of fatigue in myelofibrosis [9]. A post hoc analysis of the pivotal pacritinib trials PERSIST‐1 and PERSIST‐2 also found only modest correlations between hemoglobin improvement and decreases in fatigue; this analysis was restricted to patients with severe anemia (hemoglobin < 8 g/dL) [48]. As treatment options with potential anemia‐related benefits continue to emerge in myelofibrosis, future analyses should be designed to consider the relative impacts of specific myelofibrosis‐directed therapies on hemoglobin improvements and associated PRO changes.

As implied by the momelotinib and pacritinib analyses discussed, fatigue is a primary feature through which anemia may impact quality of life in myelofibrosis. However, fatigue in myelofibrosis is driven by factors other than anemia that may be difficult to assess; patient‐specific variables such as general activity levels and the duration of fatigue episodes experienced by a given patient must also be taken into account [2, 49, 50]. Our analyses suggest an association between anemia and fatigue through several PRO measures, both directly as part of the MPN‐SAF/MFSAF TSS and indirectly through consistent associations with related domains of general measures such as the SF‐36v2 (physical functioning and vitality domains). Notably, the association between transfusion status and the physical functioning and vitality domains of the SF‐36v2 in SIMPLIFY‐1 and SIMPLIFY‐2 was supported by similar associations with the equivalent physical functioning and fatigue domains of the EORTC QLQ‐C30 in MOMENTUM. However, the breadth of PRO measures included in this analysis also reveals associations between anemia and transfusion dependence and aspects of HRQOL beyond fatigue, including those related to general, social, and emotional health.

These findings are consistent with qualitative patient interview series that have aimed to characterize patient perceptions and impacts of anemia and transfusion dependence in myelofibrosis [16, 17, 51]. In a global study, fatigue was the most frequent symptom endorsed by patients, with weakness and night sweats also cited as common anemia‐related symptoms; the majority of patients reported associated impacts to emotional health, physical health, and social activities [16, 17]. TD patients similarly endorsed a substantial HRQOL burden related to clinical safety, convenience, and travel‐related concerns with transfusions, which impacted emotional health, physical health, and activities of daily living; symptom reduction was ranked as the most important treatment outcome, and the majority reported that treatment to control anemia and reduce transfusion burden would be beneficial [16, 17]. The findings were corroborated in an Italian study that combined quantitative modeling with qualitative interviews to assess the impact of transfusions; TD patients were estimated to spend six times more time on myelofibrosis care and incur six times greater indirect social costs than patients without anemia, with substantial work, social, and daily life impacts [51]. Collectively, this patient self‐reporting complements the quantitative clinical trial analyses described here demonstrating substantial and diverse negative associations between anemia and HRQOL in myelofibrosis, and positive associations between anemia improvement and HRQOL. Incorporation of the patient perspective would further inform development of potential novel PRO measures assessing the impact of anemia.

The main limitation of these analyses is that SIMPLIFY‐1, SIMPLIFY‐2, and MOMENTUM were not designed to prospectively evaluate the association between anemia and quality of life; these analyses are post hoc, exploratory, and descriptive. These studies were not powered for statistical comparison between groups defined by transfusion status. No formal hypothesis testing was prespecified, and the analyses were not controlled for multiplicity. The strength of conclusions that can be drawn from these analyses is also limited by the fact that the SDs were high, suggesting a high degree of uncertainty around the means. Patients receiving transfusions during the 24 week treatment period were not excluded from these analyses; thus, the impacts of continued or increased transfusion burden on both hemoglobin improvement (likely positive) or HRQOL (likely negative) are not evaluable. Data were pooled across treatment arms and trials to increase sample size; evaluation of the impact of potential cross‐trial patient population differences (e.g., JAK inhibitor exposure, anemia severity) as well as patient subgroups is precluded by sample size limitations and must await further study. In general, future studies designed to prospectively evaluate the impact of anemia on quality of life in myelofibrosis are warranted to confirm and expand on these results.

Overall, these results provide preliminary evidence of the positive association between reduced transfusion burden and hemoglobin improvement on HRQOL and symptoms in myelofibrosis, highlighting the potential value of treatments with anemia‐related benefits in improving the patient experience in myelofibrosis. While these analyses illustrate a positive association between anemia‐related benefits and HRQOL and symptoms, the high degree of uncertainty around the means suggests that currently available PRO measures largely do not adequately characterize anemia burden. In addition, future analyses are required to further elucidate the impact of anemia on other outcomes, such as survival, for a more comprehensive understanding of its role in patients with myelofibrosis. Such analyses may benefit from a threshold approach (e.g., achievement of hemoglobin > 10 g/dL in patients who had levels < 10 g/dL at baseline) rather than the point estimate approach employed here. The design and prospective evaluation of novel myelofibrosis‐specific PRO measures that better capture the effects of anemia would help to further support these efforts in prioritizing anemia management as a treatment goal to improve patient outcomes.

## Author Contributions

Conceptualization, Formal Analysis: Flora Mazerolle, Manal M'hari, Tom Liu, Shiyuan Zhang, Anna Cardellino, Zhaohui Wang, Catherine E. Ellis, Dwaipayan Patnaik, Antoine Regnault; Investigation, Resources: Ruben Mesa, Jeanne M. Palmer, Francesca Palandri, Lucia Masarova, Claire N. Harrison, Thomas W. LeBlanc; Writing: Ruben Mesa, Jeanne M. Palmer, Francesca Palandri, Lucia Masarova, Claire N. Harrison, Flora Mazerolle, Manal M'hari, Tom Liu, Shiyuan Zhang, Anna Cardellino, Zhaohui Wang, Catherine E. Ellis, Dwaipayan Patnaik, Antoine Regnault, Thomas W. LeBlanc.

## Funding

This work was supported by GSK.

## Ethics Statement

The clinical trials on which these post hoc analyses were based were conducted in accordance with the Declaration of Helsinki and Good Clinical Practice. The study protocols were approved by the institutional review board or independent ethics committee at each study site.

## Consent

All patients provided written informed consent before participation in the clinical trials on which these post hoc analyses were based.

## Conflicts of Interest

R.M. reports consulting fees from AbbVie, Blueprint, BMS, CTI BioPharma, Genentech, Geron, GSK, Incyte, MorphoSys, Novartis, and Sierra Oncology. J.M.P. reports honoraria from CTI BioPharma and advisory board participation with MorphoSys. F.P. reports consulting fees from AbbVie, Amgen, AOP Health, Celgene, CTI BioPharma, GSK, Grifols USA LLC, Karyopharm Therapeutics Inc., MorphoSys, Novartis, Sierra Oncology, and Sobi Inc. L.M. reports advisory board participation with Cogent, GSK, MorphoSys, and PharmaEssentia. C.N.H. reports institutional research funding from BMS/Celgene, Constellation Pharmaceuticals Inc. (a MorphoSys Company), and Novartis; consulting fees from AOP, Galecto, GSK, Keros, Roche, and Sobi Inc.; an advisory role and speaker funding from AbbVie, AOP Pharma, BMS/Celgene, Constellation Pharmaceuticals Inc. (a MorphoSys Company), CTI BioPharma, Galecto, Geron, GSK, Jannsen, Novartis, Promedior, and Roche; and support from Novartis for attending meetings. F.M., M.M., and A.R. report employment with Modus Outcomes, which received funding from GSK to conduct the present analysis. T.L., S.Z., A.C., Z.W., C.E.E., and D.P. report employment with and stock/stock options in GSK. T.W.L. reports institutional grants/contracts from AbbVie, American Cancer Society, AstraZeneca, BMS, Deverra Therapeutics, GSK, Jazz Pharmaceuticals, The Leukemia & Lymphoma Society, and National Institute of Nursing Research/National Institutes of Health; royalties/licenses from UpToDate; consulting fees from AbbVie, Agilix, Agios/Servier, Astellas, AstraZeneca, BeiGene, BMS/Celgene, Genentech, GSK, Lilly, Novartis, Pfizer, and Taiho; payment or honoraria from AbbVie, Agios, Astellas, BMS/Celgene, Incyte, Menarini‐Stemline, and Rigel; and stock/stock options in Dosentrx and Thyme Care.

## Supporting information


**Table S1:** PRO measures and MCTs.
**Table S2:** Mean change from baseline at week 24 in SF‐36v2 scores in the baseline TD patient subgroups in SIMPLIFY‐1 and SIMPLIFY‐2 (individual trials).
**Table S3:** Baseline characteristics of the analysis set for hemoglobin improvement.
**Table S4:** Hemoglobin improvement at week 24 in the analysis sets.
**Table S5:** Mean change from baseline in EQ‐5D‐5L index and VAS scores at week 24 based on hemoglobin improvement in SIMPLIFY‐1, SIMPLIFY‐2, and MOMENTUM (individual trials).
**Table S6:** Multivariate analysis for change from baseline in EQ‐5D‐5L index scores (dependent variable) with hemoglobin improvement at week 24 and baseline characteristics as independent variables (pooled SIMPLIFY‐1, SIMPLIFY‐2, MOMENTUM).
**Table S7:** Multivariate analysis for change from baseline in MPN‐SAF v2.0 TSS (dependent variable) with hemoglobin improvement at week 24 and baseline characteristics as independent variables (pooled SIMPLIFY‐1, SIMPLIFY‐2).
**Table S8:** Multivariate analysis for change from baseline MFSAF v4.0 TSS (dependent variable) with hemoglobin improvement at week 24 and baseline characteristics as independent variables (MOMENTUM).
**Figure S1:** EORTC QLQ‐C30 scores at baseline (A) and week 24 (B) by transfusion status at each time point in MOMENTUM.
**Figure S2:** Scores at baseline and week 24 in baseline TD patients based on SF‐36v2 in SIMPLIFY‐1 and SIMPLIFY‐2 (A) and based on EORTC QLQ‐C30 in MOMENTUM (B).

## Data Availability

Data are available upon reasonable request. Information on GSK's data‐sharing commitments and requesting access to anonymized individual participant data and associated study documents can be found at https://www.gsk‐studyregister.com/en/.
