# Supplementary material for: Association Between Transfusion Status, Hemoglobin Levels, and Patient‐Reported Outcomes in Myelofibrosis: A Post Hoc Clinical Trial Analysis
Source: Cancer Med. 2026 Apr 10;15(4):e71729. doi: 10.1002/cam4.71729 (PMC13066907; doi:10.1002/cam4.71729)
Supplement: Supplementary file 1 — Table S1: PRO measures and MCTs. Table S2: Mean change from baseline at week 24 in SF‐36v2 scores in the baseline TD patient subgroups in SIMPLIFY‐1 and SIMPLIFY‐2 (individual trials). Table S3: Baseline characteristics of the analysis set for hemoglobin improvement. Table S4: Hemoglobin improvement at week 24 in the analysis sets. Table S5: Mean change from baseline in EQ‐5D‐5L index and VAS scores at week 24 based on hemoglobin improvement in SIMPLIFY‐1, SIMPLIFY‐2, and MOMENTUM (individual trials). Table S6: Multivariate analysis for change from baseline in EQ‐5D‐5L index scores (dependent variable) with hemoglobin improvement at week 24 and baseline characteristics as independent variables (pooled SIMPLIFY‐1, SIMPLIFY‐2, MOMENTUM). Table S7: Multivariate analysis for change from baseline in MPN‐SAF v2.0 TSS (dependent variable) with hemoglobin improvement at week 24 and baseline characteristics as independent variables (pooled SIMPLIFY‐1, SIMPLIFY‐2). Table S8: Multivariate analysis for change from baseline MFSAF v4.0 TSS (dependent variable) with hemoglobin improvement at week 24 and baseline characteristics as independent variables (MOMENTUM). Figure S1: EORTC QLQ‐C30 scores at baseline (A) and week 24 (B) by transfusion status at each time point in MOMENTUM. Figure S2: Scores at baseline and week 24 in baseline TD patients based on SF‐36v2 in SIMPLIFY‐1 and SIMPLIFY‐2 (A) and based on EORTC QLQ‐C30 in MOMENTUM (B). [file CAM4-15-e71729-s001.docx]

**Supporting Information**

**Supplementary Table 1.** PRO measures and MCTs

| **SIMPLIFY-1, SIMPLIFY-2, and MOMENTUM** | | |
| --- | --- | --- |
| **EQ-5D-5L^1,2^** | **Scale** | **MCT** |
|  | Index | >0.037-0.069 |
|  | VAS | >7 |
|  | | |
| **MPN-SAF v2.0 (SIMPLIFY-1/SIMPLIFY-2)/MFSAF v4.0 (MOMENTUM)** | TSS composed of tiredness, early satiety, abdominal discomfort, night sweats, itching, bone pain, pain under ribs on left side | **MCT** |
|  |  | 1.5-2.0^a^ |
|  | | |
| **SIMPLIFY-1 and SIMPLIFY-2 only** | | |
| **SF-36v2^3,b^** | **Domain (range of possible scores, lowest-highest)** | **MCT** |
|  | Physical functioning (19.3-57.5) | ≥3.0 |
|  | Role-physical (21.2-57.2) | ≥3.0 |
|  | Bodily pain (21.7-62.0) | ≥3.0 |
|  | General health (19.0-66.5) | ≥2.0 |
|  | Vitality (22.9-70.4) | ≥2.0 |
|  | Social functioning (17.2-57.3) | ≥3.0 |
|  | Role-emotional (14.4-56.2) | ≥4.0 |
|  | Mental health (11.6-64.0) | ≥3.0 |
|  | | |
| **PGIC** | **Response** | **Score** |
|  | Very much improved | 1 |
|  | Much improved | 2 |
|  | Somewhat improved | 3 |
|  | No change | 4 |
|  | Somewhat worse | 5 |
|  | Much worse | 6 |
|  | Very much worse | 7 |
|  | | |
| **MOMENTUM only** | | |
| **EORTC QLQ-C30^4,5,c^** | **Domain** | **MCT** |
|  | Cognitive functioning, physical functioning | >7.0 |
|  | Global quality of life, social functioning | >8.0 |
|  | Emotional functioning, nausea and vomiting, dyspnea, fatigue, insomnia, pain | >9.0 |
|  | Constipation | >10.0 |
|  | Diarrhea | >11.0 |
|  | Role-functioning | >12.0 |
|  | Appetite loss | >13.0 |

EORTC QLQ-C30, European Organisation for Research and Treatment of Cancer Quality of Life Questionnaire–Core 30; MCT, meaningful change threshold; MFSAF, Myelofibrosis Symptom Assessment Form; MPN-SAF, Myeloproliferative Neoplasm Symptom Assessment Form; PGIC, Patient Global Impression of Change; PRO, patient-reported outcome; SF-36v2, 36-Item Short Form Survey, version 2; TSS, Total Symptom Score; VAS, visual analog scale.

^a^ Determined using the MFSAF v4.0.^6^ ^b^ Highest and lowest observed scores in 2009 general population normative sample. ^c^ All domains are scored on a scale from 1 to 100.

**Supplementary Table 2.** Mean change from baseline at week 24 in SF-36v2 scores in the baseline TD patient subgroups in SIMPLIFY-1 and SIMPLIFY-2 (individual trials). MCTs are 2.0 for vitality, 4.0 for role-emotional, and 3.0 for remaining domains.

| **SIMPLIFY-1 (n=73)** | **Week 24 status** | | |
| --- | --- | --- | --- |
| **Change from baseline, mean (SD)** | **Remained TD**  **(n=44)** | **Became TR**  **(n=7)** | **Became TI**  **(n=22)** |
| **Physical functioning** | 2.0 (6.0) | 7.4 (6.9) | 6.4 (6.6) |
| **Role-physical** | 2.2 (7.5) | 4.8 (5.4) | 2.0 (6.7) |
| **Mental health** | 3.6 (7.8) | −1.1 (11.6) | 5.8 (8.5) |
| **Social functioning** | −0.2 (9.4) | 4.3 (10.2) | 6.4 (7.1) |
| **Vitality** | 4.5 (8.7) | 5.1 (13.4) | 4.2 (8.9) |
|  | | | |
| **SIMPLIFY-2 (n=36)** | **Week 24 status** | | |
| **Change from baseline, mean (SD)** | **Remained TD**  **(n=17)** | **Became TR**  **(n=7)** | **Became TI**  **(n=12)** |
| **Physical functioning** | −1.1 (5.5) | 3.3 (4.4) | 2.1 (10.1) |
| **Role-physical** | −1.9 (5.0) | −2.3 (8.3) | 2.6 (9.2) |
| **Mental health** | −3.7 (8.6) | −1.5 (6.6) | −0.9 (8.1) |
| **Social functioning** | −0.9 (9.9) | −2.2 (4.9) | 2.5 (8.1) |
| **Vitality** | −2.1 (8.1) | 2.5 (10.2) | −0.5 (8.5) |

MCT, meaningful change threshold; SD, standard deviation; SF-36v2, 36-Item Short Form Survey, version 2; TD, transfusion dependent; TI, transfusion independent; TR, transfusion requiring.

**Supplementary Table 3.** Baseline characteristics of the analysis set for hemoglobin improvement

| **Characteristic^a^** | **Pooled anemic population**  **(n=480)** |
| --- | --- |
| **Age, median (range), years** | 70.0 (25-92) |
| **Male sex at birth, n (%)** | 299 (62) |
| **White, n (%)** | 390 (81) |
| **Geographic region, n (%)**  Western Europe  Eastern Europe  North America  Asia  Australasia | 228 (48)  102 (21)  99 (21)  32 (7)  19 (4) |
| **MF subtype, n (%)**  PMF  PET-MF  PPV-MF | 305 (64)  98 (20)  77 (16) |
| ***JAK2* V617F mutation positive, n (%)** | 313 (65) |
| **Platelet counts ≤150×10^9^/L, n (%)** | 250 (52) |

JAK, Janus kinase; MF, myelofibrosis; PET-MF, post–essential thrombocythemia myelofibrosis; PMF, primary myelofibrosis; PPV-MF, post–polycythemia vera myelofibrosis.

^a^ Characteristics shown are those included in multivariate analyses.

**Supplementary Table 4.** Hemoglobin improvement at week 24 in the analysis sets^a^

| **Hb improvement, n (%)** | **All 3 trials**  **(n=436)** | **SIMPLIFY-1 + SIMPLIFY-2**  **(n=241)** | **MOMENTUM**  **(n=195)** |
| --- | --- | --- | --- |
| **≥1 g/dL**  Yes  No | 193 (44)  243 (56) | 102 (42)  139 (58) | 91 (47)  104 (53) |
| **≥1.5 g/dL**  Yes  No | 134 (31)  302 (69) | 67 (28)  174 (72) | 67 (34)  128 (66) |
| **≥2 g/dL**  Yes  No | 89 (20)  347 (80) | 38 (16)  203 (84) | 51 (26)  144 (74) |

Hb, hemoglobin; PRO, patient-reported outcome.

^a^ Sample sizes for each PRO measure based on hemoglobin improvement were determined by whether the assessment was administered in all 3 trials, SIMPLIFY-1 and SIMPLIFY-2 only, or MOMENTUM only.

**Supplementary Table 5.** Mean change from baseline in EQ-5D-5L index and VAS scores at week 24 based on hemoglobin improvement in SIMPLIFY-1, SIMPLIFY-2, and MOMENTUM (individual trials). MCTs are 0.037 for index and 7 for VAS.

| **SIMPLIFY-1 (n=126)** | **Hb improvement**  **≥1 g/dL** | | **Hb improvement**  **≥1.5 g/dL** | | **Hb improvement**  **≥2 g/dL** | |
| --- | --- | --- | --- | --- | --- | --- |
| **Change from baseline, mean (SD)** | **Yes**  **(n=54)** | **No**  **(n=72)** | **Yes**  **(n=38)** | **No**  **(n=88)** | **Yes**  **(n=21)** | **No**  **(n=105)** |
| **Index** | 0.07 (0.21) | 0.06 (0.16) | 0.07 (0.22) | 0.06 (0.16) | 0.10 (0.23) | 0.06 (0.17) |
| **VAS** | 10.8 (18.2) | 4.5  (22.1) | 11.3 (19.2) | 5.4  (21.1) | 10.8 (20.0) | 6.5  (20.8) |
|  | | | | | | |
| **SIMPLIFY-2 (n=50)** | **Hb improvement**  **≥1 g/dL** | | **Hb improvement**  **≥1.5 g/dL** | | **Hb improvement**  **≥2 g/dL** | |
| **Change from baseline, mean (SD)** | **Yes**  **(n=18)** | **No**  **(n=32)** | **Yes**  **(n=11)** | **No**  **(n=39)** | **Yes**  **(n=5)** | **No**  **(n=45)** |
| **Index** | −0.03 (0.16) | −0.05 (0.17) | −0.03 (0.14) | −0.05 (0.17) | −0.01 (0.10) | −0.05 (0.17) |
| **VAS** | 4.9  (14.8) | 3.1  (23.8) | 8.0  (17.2) | 2.6  (21.8) | 19.0 (16.6) | 2.1  (20.7) |
|  | | | | | | |
| **MOMENTUM (n=121)** | **Hb improvement**  **≥1 g/dL** | | **Hb improvement**  **≥1.5 g/dL** | | **Hb improvement**  **≥2 g/dL** | |
| **Change from baseline, mean (SD)** | **Yes**  **(n=69)** | **No**  **(n=52)** | **Yes**  **(n=47)** | **No**  **(n=74)** | **Yes**  **(n=35)** | **No**  **(n=86)** |
| **Index** | 0.08  (0.27) | 0.01 (0.26) | 0.07 (0.29) | 0.03 (0.25) | 0.07 (0.31) | 0.04 (0.25) |
| **VAS** | 10.6 (20.3) | 4.9  (18.4) | 11.8 (20.9) | 5.8  (18.7) | 11.4 (22.1) | 6.8  (18.6) |

Hb, hemoglobin; MCT, meaningful change threshold; SD, standard deviation; VAS, visual analog scale.

**Supplementary Table 6.** Multivariate analysis for change from baseline in EQ-5D-5L index scores (dependent variable) with hemoglobin improvement at week 24 and baseline characteristics as independent variables (pooled SIMPLIFY-1, SIMPLIFY-2, MOMENTUM)

| **Hb improvement (n=297):** | | **≥1 g/dL**  **(yes, n=141;**  **no, n=156)** | **≥1.5 g/dL**  **(yes, n=96;**  **no, n=201)** | **≥2 g/dL**  **(yes, n=61;**  **no, n=236)** |
| --- | --- | --- | --- | --- |
| **Independent variable** | **Category** | **Parameter estimates** | | |
| **Hb improvement (ref: no)** | Yes | 0.04 | 0.03 | 0.04 |
| **Age (continuous)** | – | −0.00 | −0.00 | −0.00 |
| **Sex (ref: male)** | Female | **−0.07*** | **−0.07*** | **−0.07*** |
| **Race (ref: Black)** | Asian  Not reported  Other  White | −0.01  −0.22  −0.06  −0.13 | −0.02  **−0.23***  −0.06  −0.13 | −0.03  **−0.23***  −0.07  −0.14 |
| **Region (ref: North America)** | Asia  Australasia  Eastern Europe  Western Europe | −0.04  0.04  0.04  0.02 | −0.03  0.04  0.04  0.02 | −0.03  0.04  0.04  0.02 |
| **MF subtype (ref: primary)** | PET  PPV | 0.03  −0.03 | 0.02  −0.03 | 0.02  −0.03 |
| ***JAK2* V617F mutation status (ref: negative)** | Positive  Unknown | 0.02  −0.01 | 0.02  −0.02 | 0.02  −0.02 |
| **Baseline platelet count**  **(ref: ≤150×10^9^/L)** | >150×10^9^/L | 0.04 | 0.04 | 0.04 |
| **Baseline index score (continuous)** | – | **−0.48*** | **−0.48*** | **−0.48*** |

Parameter estimates are the predicted changes from baseline based on a given independent variable after controlling for all other independent variables. Age and baseline score were assessed as continuous variables; all others were categorical vs the indicated reference (ref) categories. Bold numbers with * indicate statistically significant changes from baseline (*P*<.05).

Hb, hemoglobin; JAK, Janus kinase; MF, myelofibrosis; PET, post–essential thrombocythemia; PPV, post–polycythemia vera.

**Supplementary Table 7.** Multivariate analysis for change from baseline in MPN-SAF v2.0 TSS (dependent variable) with hemoglobin improvement at week 24 and baseline characteristics as independent variables (pooled SIMPLIFY-1, SIMPLIFY-2)

| **Hb improvement (n=297):** | | **≥1 g/dL**  **(yes, n=141;**  **no, n=156)** | **≥1.5 g/dL**  **(yes, n=96;**  **no, n=201)** | **≥2 g/dL**  **(yes, n=61;**  **no, n=236)** |
| --- | --- | --- | --- | --- |
| **Independent variable** | **Category** | **Parameter estimates** | | |
| **Hb improvement (ref: no)** | Yes | −0.74 | −0.44 | −1.91 |
| **Age (continuous)** | – | 0.07 | 0.07 | 0.08 |
| **Sex (ref: male)** | Female | 1.50 | 1.49 | 1.47 |
| **Race (ref: Black)** | Asian  Not reported  Other  White | −2.94  5.11  −3.00  3.48 | −2.71  5.45  −3.14  3.78 | −1.87  5.77  −1.87  4.25 |
| **Region (ref: North America)** | Asia  Australasia  Eastern Europe  Western Europe | 2.64  3.00  −0.64  1.67 | 2.82  2.99  −0.60  1.65 | 2.59  3.18  −0.61  1.79 |
| **MF subtype (ref: primary)** | PET  PPV | 1.32  −0.48 | 1.26  −0.59 | 1.32  −0.55 |
| ***JAK2* V617F mutation status (ref: negative)** | Positive | −0.45 | −0.34 | −0.42 |
| **Baseline platelet count**  **(ref: ≤150×10^9^/L)** | >150×10^9^/L | −0.96 | −0.97 | −1.09 |
| **Baseline TSS (continuous)** | – | **−0.29*** | **−0.29*** | **−0.29*** |

Parameter estimates are the predicted changes from baseline based on a given independent variable after controlling for all other independent variables. Age and baseline score were assessed as continuous variables; all others were categorical vs the indicated reference (ref) categories. Bold numbers with * indicate statistically significant changes from baseline (*P*<.05).

Hb, hemoglobin; JAK, Janus kinase; MF, myelofibrosis; MPN-SAF, Myeloproliferative Neoplasm Symptom Assessment Form; PET, post–essential thrombocythemia; PPV, post–polycythemia vera; TSS, Total Symptom Score.

**Supplementary Table 8.** Multivariate analysis for change from baseline MFSAF v4.0 TSS (dependent variable) with hemoglobin improvement at week 24 and baseline characteristics as independent variables (MOMENTUM)

| **Hb improvement (n=297):** | | **≥1 g/dL**  **(yes, n=141;**  **no, n=156)** | **≥1.5 g/dL**  **(yes, n=96;**  **no, n=201)** | **≥2 g/dL**  **(yes, n=61;**  **no, n=236)** |
| --- | --- | --- | --- | --- |
| **Independent variable** | **Category** | **Parameter estimates** | | |
| **Hb improvement (ref: no)** | Yes | **−4.25*** | −3.65 | −2.59 |
| **Age (continuous)** | – | **−0.30*** | **−0.30*** | **−0.29*** |
| **Sex (ref: male)** | Female | 0.74 | 0.91 | 0.79 |
| **Race (ref: Black)** | Asian  Other  White | 3.57  5.45  8.22 | 2.31  4.25  6.65 | 1.34  3.79  5.63 |
| **Region (ref: North America)** | Australasia  Eastern Europe  Western Europe | **−11.66***  −2.30  −5.45 | −10.08  −2.03  −4.70 | −9.91  −2.12  −4.91 |
| **MF subtype (ref: primary)** | PET  PPV | −2.32  −0.93 | −1.87  −1.17 | −1.79  −1.19 |
| ***JAK2* V617F mutation status (ref: negative)** | Positive  Unknown | −2.15  −8.52 | −2.16  −7.50 | −2.15  −6.84 |
| **Baseline platelet count**  **(ref: ≤150×10^9^/L)** | >150×10^9^/L | 1.03 | 0.91 | 1.32 |
| **Baseline TSS (continuous)** | – | **−0.60*** | **−0.61*** | **−0.61*** |

Parameter estimates are the predicted changes from baseline based on a given independent variable after controlling for all other independent variables. Age and baseline score were assessed as continuous variables; all others were categorical vs the indicated reference (ref) categories. Bold numbers with * indicate statistically significant changes from baseline (*P*<.05).

Hb, hemoglobin; JAK, Janus kinase; MF, myelofibrosis; MFSAF, Myelofibrosis Symptom Assessment Form; PET, post–essential thrombocythemia; PPV, post–polycythemia vera; TSS, Total Symptom Score.

**Supplementary Figure 1. EORTC QLQ-C30 scores at baseline (A) and week 24 (B) by transfusion status at each time point in MOMENTUM.** Higher scores indicate improvement for all 4 functioning domains but worsening for the fatigue domain. EORTC QLQ-C30, European Organisation for Research and Treatment of Cancer Quality of Life Questionnaire–Core 30; SD, standard deviation; TD, transfusion dependent; TI, transfusion independent; TR, transfusion requiring.

A. Baseline


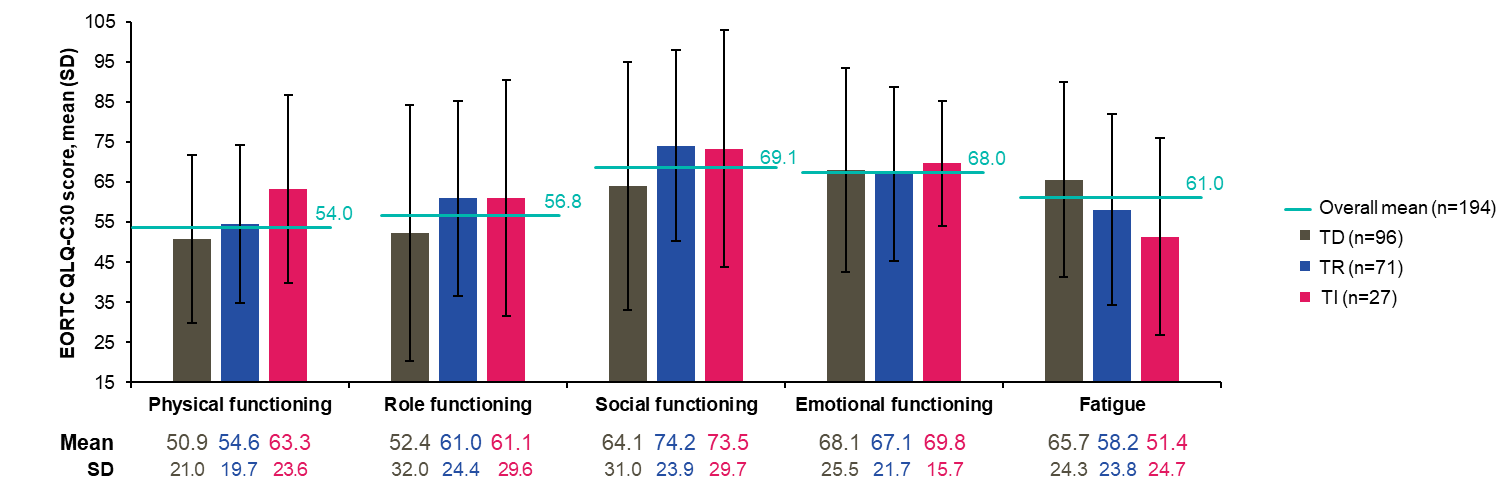


B. Week 24


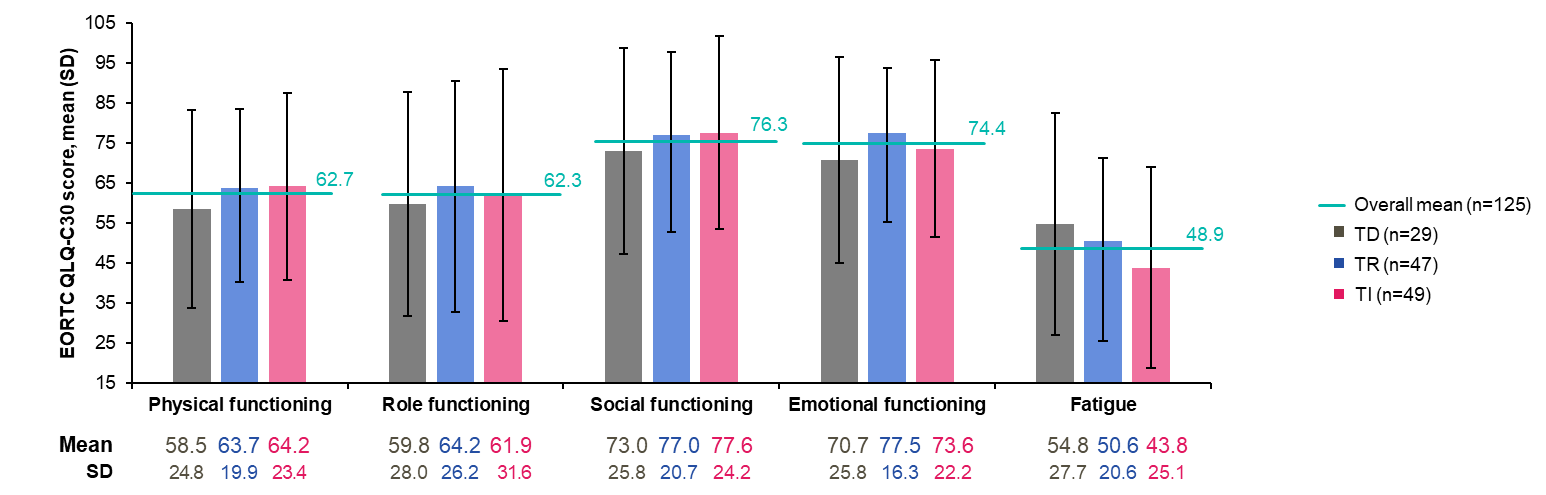


**Supplementary Figure 2. Scores at baseline and week 24 in baseline TD patients based on SF-36v2 in SIMPLIFY-1 and SIMPLIFY-2 (A) and based on EORTC QLQ-C30 in MOMENTUM.** The left bar for each domain indicates the distribution of scores at baseline in the subgroup of patients who were TD, while the other 3 indicate the distributions at week 24 for patients in that subgroup who remained TD, became TR, or became TI, respectively. EORTC QLQ-C30, European Organisation for Research and Treatment of Cancer Quality of Life Questionnaire–Core 30; NBS, norm-based score; SD, standard deviation; SF-36v2, 36-Item Short Form Survey, version 2; TD, transfusion dependent; TI, transfusion independent; TR, transfusion requiring.

A. SIMPLIFY-1/SIMPLIFY-2


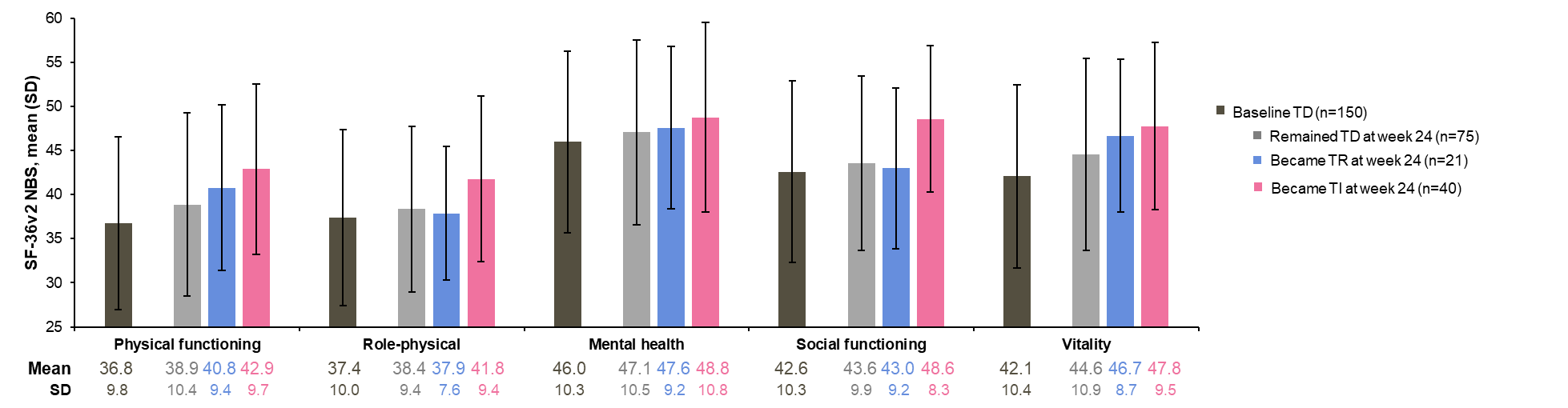


B. MOMENTUM

**
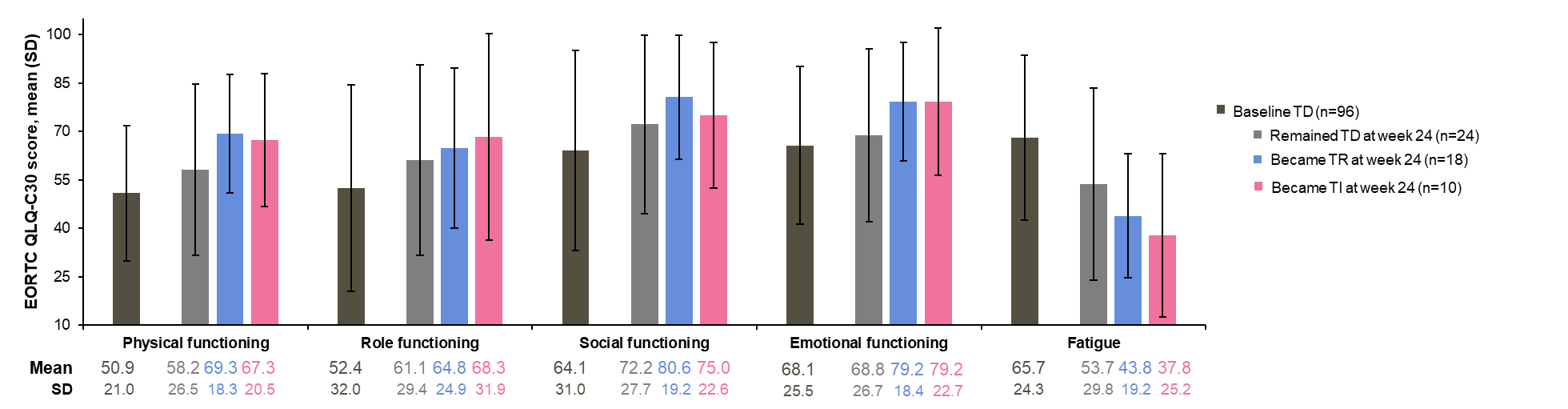
**

**Supplementary References**

1. Pickard AS, Neary MP, Cella D. Estimation of minimally important differences in EQ-5D utility and VAS scores in cancer. *Health Qual Life Outcomes.* 2007;5:70.

2. McClure NS, Sayah FA, Xie F, Luo N, Johnson JA. Instrument-defined estimates of the minimally important difference for EQ-5D-5L index scores. *Value Health.* 2017;20(4):644-650.

3. Mariush ME, ed. *User’s Manual for the SF-36v2 Health Survey*. 3rd ed. QualityMetric Incorporated; 2011.

4. Mesa RA, Harrison C, Palmer JM, et al. Patient-reported outcomes and quality of life in anemic and symptomatic patients with myelofibrosis: results from the MOMENTUM study. *Hemasphere.* 2023;7(11):e966

5. Cocks K, King MT, Velikova G, et al. Evidence-based guidelines for interpreting change scores for the European Organisation for the Research and Treatment of Cancer Quality of Life Questionnaire Core 30. *Eur J Cancer.* 2012;48(11):1713-1721.

6. Mesa R, et al. Assessment of minimal clinically important difference in patient-reported myelofibrosis-associated symptoms using an anchor-based analysis based on MANIFEST arm 3 data. Presented at: 65th ASH Annual Meeting and Exposition; December 9-12, 2023; San Diego, CA. Poster 3195.
